# ‘Brünnerling’ group show that close relatedness and polyploidy make apple genetic diversity highly complex

**DOI:** 10.1186/s13104-026-07786-7

**Published:** 2026-03-27

**Authors:** Lea Broschewitz, Hans-Joachim Bannier, Monika Höfer, Henryk Flachowsky

**Affiliations:** 1 Julius Kühn Institute (JKI), Federal Research Centre for Cultivated Plants, Institute for Breeding Research on Fruit Crops, Pillnitzer Platz 3a, 01326 Dresden-Pillnitz, Germany; 2 Pomologen-Verein e.V, Husumer Straße 16, 20251 Hamburg, Germany

**Keywords:** Pomology, Genetic fingerprint, Parentage analysis, Literature research

## Abstract

**Objective:**

This study uses molecular marker data from the apple cultivar collection of the German Fruit Genebank (GFG) to test the genetic coherence of the literature-defined ‘Brünnerling’ group and to resolve mislabelling and relationships through kinship/parentage and genetic distance analyses.

**Results:**

Parentage and genetic distance-based analyses showed that ‘Brünnerling’ cultivars can be distinguished on the molecular level. ‘Kleiner Brünnerling’ (Malus UNiQue genotype code (MUNQ) 2284) was identified as a major progenitor within the cultivar group. Pedigree relationships among the ‘Brünnerling’ cultivars are complex, particularly for triploid cultivars. Parentages from previous studies were confirmed with regard to MUNQs. For the putative triploid cultivar ‘Bühlers Erdbeerapfel’ (MUNQ 898), the genetic fingerprint data corresponded to the transfer of an unreduced gamete from MUNQ 8143, which is one of the two parental genotypes. In addition, it was also possible to identify the second parent. In the distance-based tree, the ‘Brünnerling’ cultivars formed a cluster with other cultivars, and the central role of ‘Kleiner Brünnerling’ was further highlighted.

**Supplementary Information:**

The online version contains supplementary material available at 10.1186/s13104-026-07786-7.

## Introduction

Historically, apple (*Malus ssp*.) cultivars are classified solely based on the phenotypic characteristics of their fruit. In recent years, genetic relationships were incorporated into classification systems. One group of cultivars that is a good example of the complex diversity of apple is the ‘Brünnerling’ group [1]. This group consists of some of the most important cultivars of the Alpine region. These cultivars were first mentioned in the 17th century, and they have since been seen as a “national fruit” of different regions of the historic Habsburg Empire. From nowadays Austria, the distribution stretched throughout Switzerland and to the south of Germany. Across the historic, German literature, cultivars of the ‘Brünnerling’ group were described under different (sometimes misleading) names [1–6]. In some cases, names were adopted incorrectly or simply copied for centuries [1, 3, 7–9]. This has caused difficulties when identifying cultivars and even today leads to genotypic data being associated with incorrect names. Another difficulty is the lack of continuous cultivar collections. While cultivars were known back in the day, even with reference trees, these were lost over time. This publication aims to help clear up the difficulty of relying on literature sources by integrating more objective information from molecular analysis.

The apple cultivar collection of the German Fruit Genebank (GFG; https://www.deutsche-genbank-obst.de/), which is a decentralized genebank network across Germany, was used for this study [10, 11]. This collection was previously characterized pomologically and molecularly [12–14] and was used here to investigate whether the ‘Brüünnerling’ cultivars represent a genetically coherent cultivar group, and to resolve misnaming/synonymy by reconstructing genetic relationships among candidate accessions.

## Main

### Methods

The ‘Brünnerling’ cultivars were described in (historic) literature [1–9, 15], and nowadays institutions and pomologists put in the effort to clearly distinguish them [16–20]. The gathered information from these sources (written in German) were translated (Supplementary Document 1).

The molecular data used are based on a published SSR marker dataset [14]. The sourcing of data is described in a data descriptor based on the preceding dataset [13, 21]. As the tools used in this study are limited to diploid data, only the first two alleles were kept, resulting in a forced diploid dataset even though the cultivars also express tri- and tetraploid genetic profiles. This dataset contained 1,565 apple cultivars, of which 21 were grouped into the ‘Brünnerling’ group (Table 1, Supplementary Table 1). The cultivar selection was based on input by the German pomologist Hans-Joachim Bannier [16] and the apple geneticist Nicholas P. Howard, as well as a preliminary evaluation on genetic similarity (JKI, data not shown) [22, 23]. Important were the Malus UNiQue genotype numbers (MUNQs) that enable alignment to other published studies [24, 25].

The parentage analysis was performed in two steps: (I) an unbiased maternity analysis to identify the unknown first parent, and (II) a maternity analysis, where results from the pair analysis were used to infer the second parent. The forcibly diploid dataset was used to determine the possible parentage of 20 ‘Brünnerling’ cultivars (‘Kleiner Brünnerling’ excluded). As potential parents, only (putative) diploid cultivars were tested. This analysis was performed with CERVUS v3.0.7 [26, 27]. After default allele frequency analysis, one simulation for both approaches was run with 10,000 offspring, 100,000 candidate mothers, 0.7 proportion of samples, 0.9971 proportion of loci typed, and 15 minimum typed loci. The results were reviewed manually to take into account the deleted allele variants and minimize erroneous assignments of parentage (Supplementary Table 2).

The distance-based tree was constructed in DARwin v6.0.21 [28]. The allelic data were used to calculate dissimilarities with 100 bootstraps and followed by unweighted neighbour-joining tree construction. The resulting tree was visualized in the web tool Interactive Tree of Life (iTOL) V6 [29]. The tree was re-rooted for *Malus* × *floribunda* 821, and the 21 ‘Brünnerling’ cultivars were manually annotated based on assumed ploidy levels. Ploidy level was assumed based on the complete SSR profiles [14], while only the forced diploid dataset was used for tree construction.

## Results

The parentage analysis revealed that ‘Kleiner Brünnerling’ (MUNQ 2284) is a potential parent for 17 out of 20 ‘Brünnerling’ cultivars (Table 1, Supplementary Table 1). The statistically achieved results were cross-checked manually by assigning the possible inheritance of the alleles (Supplementary Table 2). With the manual check, all alleles could be included, as some alleles were excluded in support of the forced diploid dataset. The inferred first parent for all tested ‘Brünnerling’ cultivars except ‘Bühlers Erdbeerapfel’ (MUNQ 898) is sound based on the parentage analysis and manual assessment. The full parentage for ‘Steirischer Maschanzker’ (MUNQ 264), ‘Falsche Biherol Rentte‘ (MUNQ 1267) and ‘Thurgauer Weinapfel’ (MUNQ 1333) was confirmed statistically and manually. The parentage of ‘Steirischer Maschanzker’ and ‘Thurgauer Weinapfel’ was previously investigated [22, 30]. For ‘Echter Winterstreifling’ (MUNQ 11812), four alleles were not accounted for by the suggested parentage (Supplementary Table 2). This cultivar was investigated because ‘Kleiner Brünnerling’ was suggested as one of its parents [22]. In this parentage analysis, ‘Roter von Simonffi’ (MUNQ 1377) and MUNQ 8152 are suggested as parents. The GFG accession for ‘Echter Winterstreifling’ was not confirmed to be true-to-type yet, as pomological confirmation is still missing [14]. Therefore, an erroneous cultivar name assignment cannot be excluded.

**Table 1:** Results of parentage analysis for 20 select ‘Brünnerling’ cultivars

Cultivar name offspring	MUNQ offspring	Cultivar name parent 1	MUNQ parent 1	Pair loci mismatching (offspring & parent 1)	Pair confidence (offspring & parent 1)	Cultivar name parent 2	MUNQ parent 2	Pair loci mismatching (offspring & parent 2)	Pair confidence (offspring & parent 2)	Trio confidence
Steirischer Maschanzker	264	Kleiner Brünnerling	2284	0	+	Französische Edelrenette	278	0		*
Bühlers Erdbeerapfel	898	*unknown*	*11,757*	*0*	*					
Welschisner	1051	Kleiner Brünnerling	2284	0	*	*unknown*	*8193*	*0*	*+*	*
Falsche Biherol Renette^1^	1267	Baumanns Renette	21	0	*	Kleiner Brünnerling	2284	0	*	*
Thurgauer Weinapfel	1333	Kleiner Brünnerling	2284	0	*	Fraurotacher	202	1	*	*
Brünnerling-relative 1^1^	5426	Kleiner Brünnerling	2284	0	*					
Oberösterreichischer Brünnerling	5489	Kleiner Brünnerling	2284	0	-					
Sohlander Streifling	5515	Kleiner Brünnerling	2284	0	*					
Brünnerling-relative 2^1^	6478	Kleiner Brünnerling	2284	1	*					
Malerapfel	8021	Kleiner Brünnerling	2284	0	*					
Weber Bartl	8078	Kleiner Brünnerling	2284	0	*					
Welschbrunner	8079	Kleiner Brünnerling	2284	0	*					
Bayerischer Brünnerling	8105	Kleiner Brünnerling	2284	0	*					
Winterscheibling	8119	Kleiner Brünnerling	2284	0	*					
Unknown	8143	Äckerle-Apfel	5594	0	*					
Mauthausener Limoniapfel	8168	Kleiner Brünnerling	2284	0	*	*Schmidbergers Renette*	*1015*	*3*		*+*
Brünnerling-descendent^1^	8210	Kleiner Brünnerling	2284	0	*	*Früher Isnyer*	*11,756*	*1*		*
Früher Isnyer	11,756	Kleiner Brünnerling	2284	1	*	*Hendunger Weißapfel*	*7996*	*3*		*
Unknown	11,757	Kleiner Brünnerling	2284	0	*					
Echter Winterstreifling	11,812	Roter von Simonffi	1377	0	*	*unknown*	*8152*	*2*	*-*	*

Pair & Trio Confidence: “*” for strict confidence (95%), “+” for relaxed confidence (80%), “-“ is shown for a most likely candidate parent within the dataset. More detailed results and statistics can be found in Supplementary Table 1; Manual assessment: detailed information & genetic profile comparisons can be found in Supplementary Table 2. Written in *grey italic* are the results that did not hold up to the manual check

**Fig. 1:**
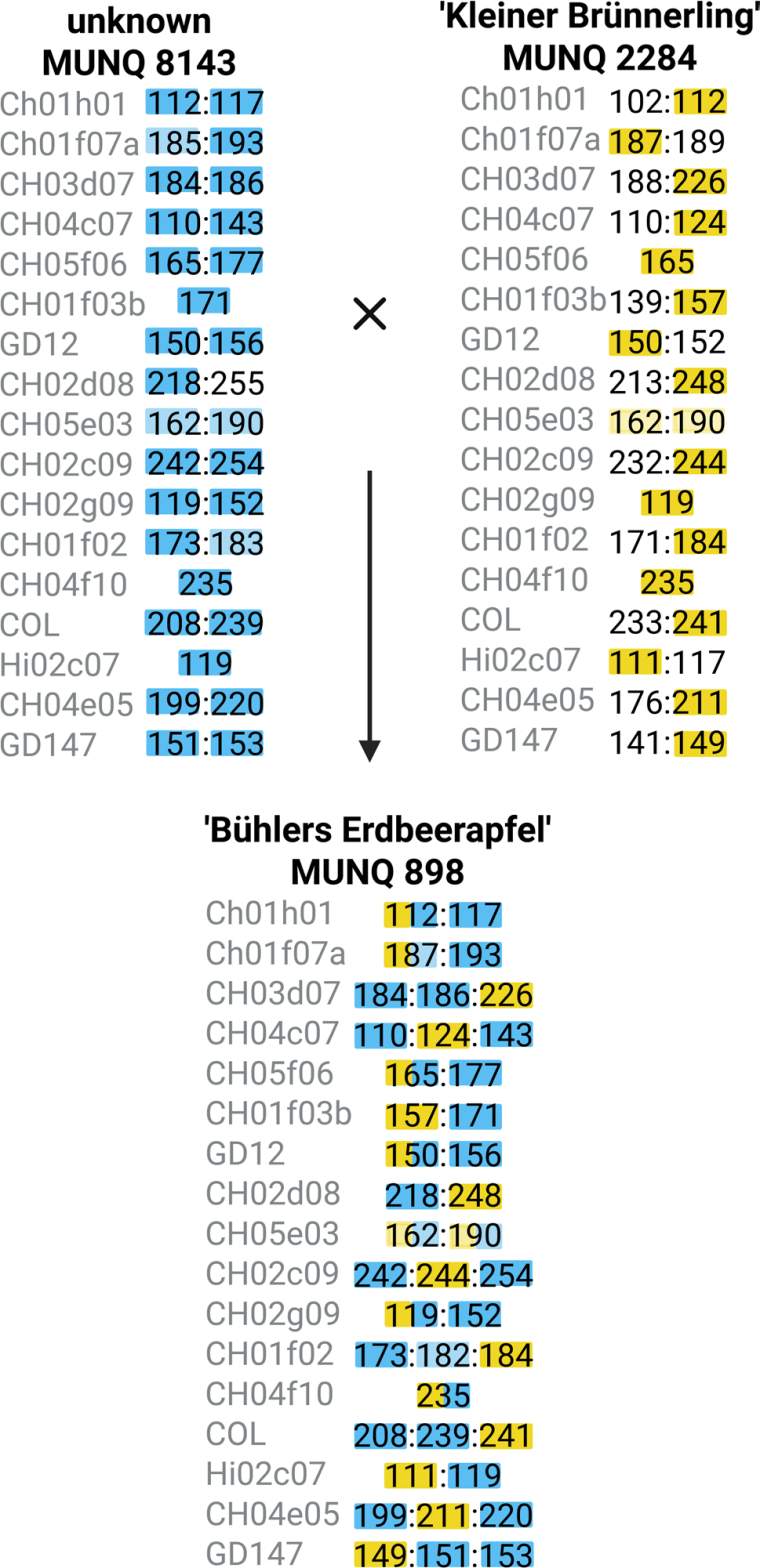
Reconstructed parentage of ‘Bühlers Erdbeerapfel’ MUNQ 898 based on allele variants of SSR marker data (Supplementary Table 2). Allele variants inherited from the first parent are highlighted in blue and alleles inherited from the second parent are highlighted in yellow. If there was some uncertainty, the colour shade is lighter. Seven markers amplify three alleles in MUNQ 898, indicating a triploid genotype. Two alleles of each of these markers originate from MUNQ 8143.The figure was created in BioRender (https://BioRender.com/idfcc6p)

For ‘Bühlers Erdbeerapfel’, MUNQ 11,757 was proposed as a parent. Manual validation does not support this relationship (Table 1). Results of a preliminary investigation tend to favour a parentage of MUNQ 8143 and ‘Kleiner Brünnerling’ (JKI, data not shown). The manual evaluation of this progeny-parent trio seems very plausible (Fig. 1, supplementary Table 2). This case is particularly interesting because ‘Bühlers Erdbeerapfel’ is probably triploid, both parents are diploid and we can infer the pedigree from the manual evaluation. Each allele is accounted for perfectly except for one possible technical shift at marker CH01f02 (Supplementary Table 2). At that locus, MUNQ 8143 expresses 183 bp while ‘Bühlers Erdbeerapfel’ expresses 182 bp, but the allele can still be considered a match. In addition, it can be observed that the unknown cultivar MUNQ 8143 inherits two alleles, while the ‘Kleiner Brünnerling’ inherits only one (Fig. 1).

In other triploid cultivars (Supplementary Table 2), the ‘Kleiner Brünnerling’ may have inherited two alleles (e.g. ‘Oberösterreichischer Brünnerling’ (MUNQ 5489), ‘Brünnerling-relative 1’ (MUNQ 5426), ‘Brünnerling-relative 2’ (MUNQ 6478), ‘Welschbrunner’ (MUNQ 8079), ‘Bayerischer Brünnerling’ (MUNQ 8105), ‘Winterscheibling’ (MUNQ 8119) and ‘Brünnerling-descendent’ (MUNQ 8210)). For these cultivars, a confident second parent could not be identified, but would greatly support this analysis if the genetic profiles align.

**Fig. 2:**
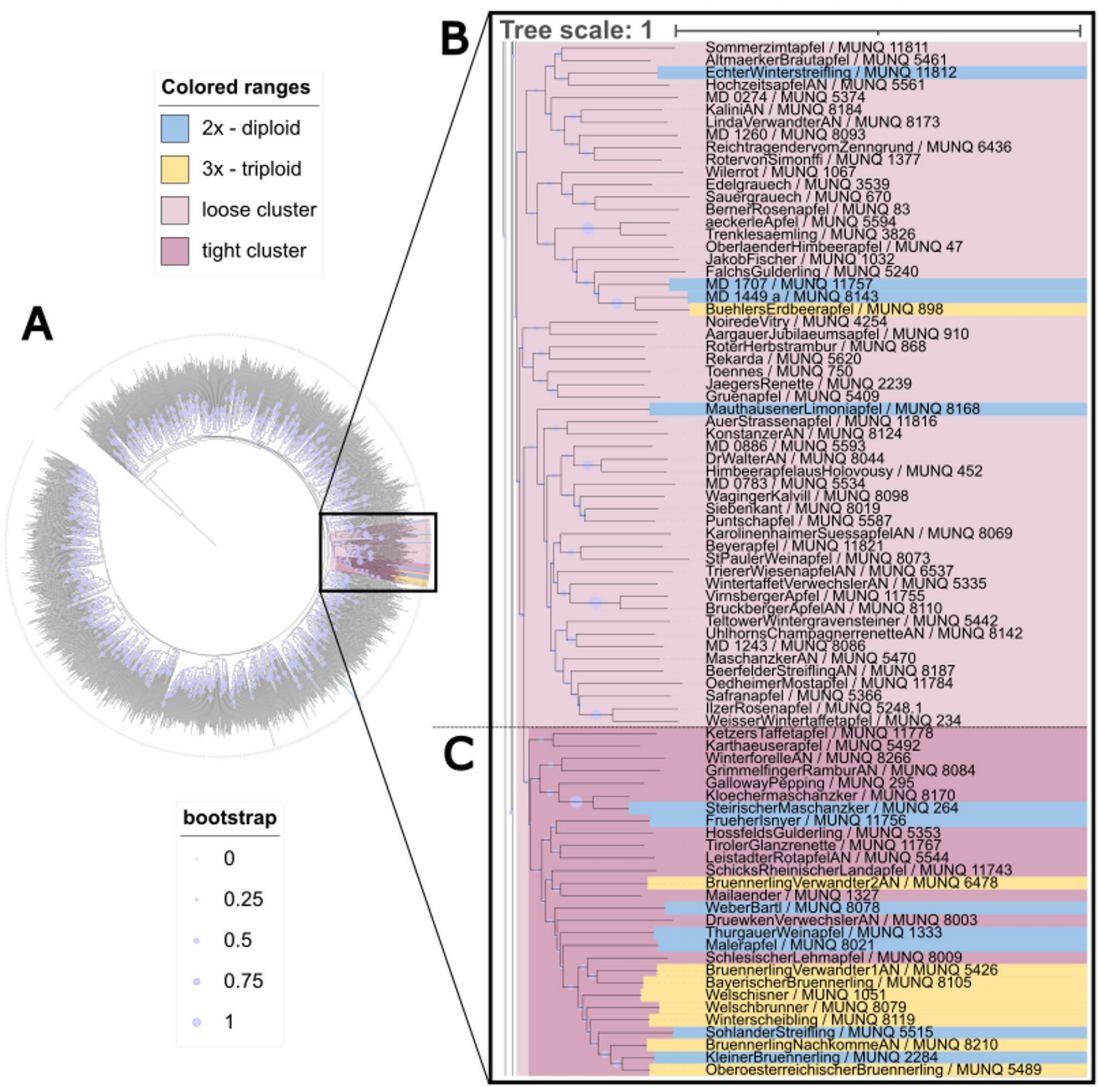
Distance-based tree illustrating similarity among apple cultivars with focus on ‘Brünnerling’ group. **A** complete tree of 1,565 apple cultivars, detailed view is available is Supplementary Fig. 1. **B** Cut-out shows a cluster where individual ‘Brünnerling’ cultivars are grouped with other cultivars and **C** sub-cluster were several ‘Brünnerling’ cultivars are grouped very closely together. Label show if a cultivar is suggested diploid (blue) or triploid (yellow). Gray sidebars in (B) indicate sub-clusters for ‘Roter von Simonffi’ and ‘Weißer Wintertaffetapfel’. Detailed information can be found in Supplementary Table 3

A distance-based tree was built with all available apple genotypes, while the focus is set on the cluster that contains the majority of ‘Brüünnerling’ cultivars (Fig. 2A). This cluster contains 83 out of the 1,565 cultivars and all but one ‘Brünnerling’ cultivar (Fig. 2B, Supplementary Table 3). ‘Falsche Biherol Renette’ cannot be found in proximity to the other ‘Brünnerling’ cultivars. Interestingly, this genotype showed ‘Kleiner Brünnerling’ as one of its parents (Table 1). In a sub-cluster, 15 of the 21 ‘Brünnerling’ cultivars can be found (Fig. 2C). The previously discussed close relationship between ‘Bühlers Erdbeerapfel’ and MUNQ 8143 as well as the close relation between ‘Kleiner Brünnerling’ and its assumed triploid progeny, is expressed by the proximity between them. In addition, there are further sub-clusters dedicated to certain “founding” cultivars based on the information obtained about possible ancestries (Fig. 2B, grey sidebars; JKI, data not shown). One sub-cluster revolves around ‘Roter von Simonffi’ and another around ‘Weißer Wintertaffetapfel’ (MUNQ 234). In both cases, many cultivars in the sub-cluster were inferred as possible offspring of the respective cultivar (JKI, data not shown).

## Discussion

Historically defined ‘Brünnerling’ cultivars were examined for their genetic coherence. In addition to the historical literature, detailed pomological investigations and genetic fingerprint analyses were carried out. The genetic fingerprints were used for genetic relationship studies and parentage analyses.

Across analyses, the genetic data support close relationships among many accessions of the ‘Brünnerling’ complex. The parentage analysis highlighted that ‘Kleiner Brünnerling’ is a major progenitor and founder cultivar of this group, as was previously suggested by Löschnig et al. (1912) [1]. In this work, we could ascertain this hypothesis by using molecular data. Furthermore, it was possible to confirm suggested parentages from other studies (Howard et al. [22], Muranty et al. [30]), as in the cases of ‘Steirischer Maschanzker’ and ‘Thurgauer Weinapfel’. From an applied genetics perspective, these cross-study highlight the need for MUNQs to harmonize datasets across institutions and countries when cultivar names differ, ensuring reliable transfer of results to breeding, association studies, and conservation decisions.

Another important finding concerns the impact of ploidy as shown for ‘Bühlers Erdbeerapfel’. Such triploids can result from diploid parents when one of the gametes is not reduced during meiosis. Unreduced gamete-donating parents were already reported as often observed in historic triploid cultivars [23]. However, triploids are rarely parents of other cultivars, as they only form fertile gametes in individual cases [23, 31, 32]. The parentage of ‘Bühlers Erdbeerapfel’ could be reconstructed. It was shown that this cultivar originates from the fertilisation of a reduced (1n) and an unreduced (2n) gamete. In other triploid ‘Brünnerling’ cultivars, the unreduced gamete apparently originated from ‘Kleiner Brünnerling’. Interestingly, there were cases for both pre- (e.g. MUNQ 5489 and MUNQ 8105) and post-meiotic (e.g. MUNQ 8119) genome duplication.

The suggestion for the pedigree of ‘Bühlers Erdbeerapfel’ relied heavily on the manual evaluation. The parentage analysis statistically did not consider ‘Kleiner Brünnerling’ as a parent for ‘Bühlers Erdbeerapfel’. The dataset was converted into a diploid dataset by removing all but the first two alleles and therefore became biased to accommodate the bioinformatic tools. The manual assessment showed that in this deletion process, three alleles of ‘Bühlers Erdbeerapfel’ were removed that are specific for ‘Kleiner Brünnerling’. This highlights the necessity for polyploid-aware workflows and targeted manual validation to avoid systematic misassignment.

The importance of the genotype similarity is further expressed in the distance-based tree. Many of the triploid ‘Brünnerling’ cultivars that have inherited two alleles from the ‘Kleiner Brünnerling’ group are closely together. Yet it was also found that many other cultivars group with ‘Brünnerling’ cultivars genetically as well. This provides a new range of cultivars that can be investigated in this context in the future. In contrast, ‘Falsche Biherol Renette’, which is a direct descendant of ‘Kleiner Brünnerling’, did not cluster closely. Supposedly, the genetic relation to ‘Baumanns Renette’ (MUNQ 21) had a stronger influence here. Robust cultivar classification benefits from triangulating evidence across marker-based clustering, pedigree reconstruction, and pomological data rather than relying on any single line of evidence.

Historical names and fruit phenotypes are insufficient to delimit cultivar groups in cases with centuries of copying and regional renaming. By integrating molecular evidence, we provide a more objective basis to (i) verify which accessions truly belong to the ‘Brünnerling’ complex, (ii) indicate misassigned names, and (iii) stabilize cultivar definitions for genebank curation and downstream genetic studies. This study uses the ‘Brünnerling’ complex as a model to demonstrate that cultivar-group definitions should be treated as testable genetic hypotheses, not solely as historically inherited phenotypic/literature categories.

### Limitations

- Many fingerprint analysis tools only support diploid data, so access alleles were removed. This proved problematic for parentage analysis of triploid cultivars, as parental alleles could be lost (e.g. ‘Bühlers Erdbeerapfel’), leading to biased statistics. This highlights the need for manual genotype assessment, while statistical approaches remain useful for large datasets or initial evaluations. Including historical literature was also valuable, particularly for developing parentage hypotheses.
- The dataset used is limited only to cultivars that were known in Germany. The ‘Brünnerling’ cultivars were possibly widespread in the former Habsburg monarchy. It is possible that there are other historical cultivars in countries of this region that are related to ‘Brünnerling’ cultivars. However, information confirming this (genetic or from pomological literature) is not yet available.
- The use of 17 SSR markers harbours the risk of reduced representation of a genotype. However, the analysis of the GFG’s apple cultivars has shown that 6 SSR’s are sufficient to determine a genotype beyond doubt [12]. More confident results for parentage analysis might be achieved with SNP marker analysis [33].

## Supplementary Information

Below is the link to the electronic supplementary material.

Supplementary Material 1.

Supplementary Material 2.

Supplementary Material 3.

## Data Availability

The data analysed during the current study are filtered from a previously published dataset freely available in the OpenAgrar repository: https://doi.org/10.5073/20250813-152720-0 [14]. Extensive description of the data sourcing is provided by the respective data descriptor: https://doi.org/10.1038/s41597-025-04390-5 [13].

## Abbreviations

GFG: German fruit genebank
MUNQ: Malus unique genotype code
SSR: Simple sequence repeat
