# Supplementary material for: ‘Brünnerling’ group show that close relatedness and polyploidy make apple genetic diversity highly complex
Source: BMC Res Notes. 2026 Mar 27;19:164. doi: 10.1186/s13104-026-07786-7 (PMC13063727; doi:10.1186/s13104-026-07786-7)
Supplement: Supplementary file 2 — Supplementary Material 2. [file 13104_2026_7786_MOESM2_ESM.docx]

# Supplemental document 1: extended literature information on the ‘Brünnerling’ group

The historic literature on ‘Brünnerling’ cltivars was critically reviewed and summarized by the pomology expert H.-J. Bannier [1]. Here, the work is summarized and translated from German into English by L. Broschewitz.

## The name ‘Brünnerling’

This cultivar ‘Prinerling’ (Brünnerling) name was first mentioned in the „Georgica Curiosa Aucta“ which dates back to 1659 [2].The origin and meaning of the term “Brünnerling” was heavily discussed. Stoll (1888) speculated the name indicates Moravia as the origin since the city of Brünn is located there [3]. Another suggestion is the derivation from the German word for fountain or well, “Brunnen”, and would thus be equivalent to the name “Brunnenapfel” (fountain apple) [3]. Löschnig (1912), however, explicitly disagreed to Stoll’s speculation on the connection to Moravia or fountains. The designation “Brünerling” or “Brinnerling” is rooted in the old German word “bruna” and the folk expression “brinnend” which can be translated to “to shine” and “glowing”, respectively [4]. This refers to the vivid red colouration on the yellow ground colour of the fruit.

## ‘Kleiner Brünnerling’

As the conservation of rare genetic resources is on the main goals of the GFG, the rediscovery of the lost cultivar ‘Kleiner Brünnerling’ was an especially lucky find. The single accession was maintained by Competence Centre of Fruit Production – Lake Constance under the wrong name of ‘Welschisner’. Accepted synonymes for this cultivar are ‘Nägeliapfel’, ‘Palmapfel’ and ‘Campanner’ [5-8].The fruit shape is oblate [4,9]. The smaller and flatter fruits are the main distinction when compared to other ‘Brünnerling’-cultivars [4,10]. The ground colour of the fruit is yellow green with red to brown red hue of over colour [4,8,9]. Compared to other ‘Brünnerling’-cultivars, the length of the stalk can also be medium length instead of primarily short. Another characteristic was the abundance of viable seeds, indicating diploid (2x) ploidy levels [5,7,8]. The ploidy level was later on confirmed by the molecular analysis [11]. The cultivar most likely originates from the larger Austrian-Czech area [10]. Internationally, this cultivar groups with MUNQ 2284 [11]. The authenticity of the cultivar with this specific MUNQ and the associated name ‘Kleiner Brünnerling’ was approved based on molecularly determined family relations by integrating information from historic literature, especially from Löschnig et al. (1912, 1948)[4,12,13]

## ‘Welschisner’

Other used names for the ‘Welschisner‘ are ‘Welsch Isnyer‘ and ‘Großer Böhmischer Brünnerling‘. Today, Bannier reports that this ‘Brünnerling’-cultivar is the most common one in Germany and the Alpine region[1]. The fruit are medium to large in size with pink red to red hue of over color on yellow green ground color [9,14,15]. Löschnig (1912) describes the ‘Böhmischer Brünerling’ as the largest ‘Brünnerling’-variety [4]. The fruit shape is oblong to conic with irregular edging [1,4,9]. The stalk cavity is unusually broad and the seed compartments of the core are relatively large and fissured [1,3]. The leaves of this cultivar are crenate/ bluntly serrated [9,14]. The GFG carries ten accessions of this cultivar in its collection. Molecular analyses showed that this cultivar is triploid (3x) and it can be identified with MUNQ 1051 [11].

## ‘Oberösterreichischer Brünnerling’

Synonyms for this cultivar are ‘Landler’ and ‘Zwiebelapfel’ [4]. The ‘Oberösterreichischer Brünnerling’ is distributed more scarcely across Austria [1,16]. The fruit shape is characterized by edges and the hue of over colour is less intense compared to ‘Kleiner Brünnerling’[4]. Compared to the ‘Welschisner’ the leaves are sharply serrate instead of crenate indicating also the importance of characteristic unrelated to the fruit in the science of pomology [9,14].In the GFG, only two accession of this cultivar are accounted for. The ‘Oberösterreichischer Brünnerling’ is a triploid cultivar and is associated with MUNQ 5489 [11].

## ‘Welschbrunner’

This cultivar is mainly spread across eastern Austria and Styria [4]. In these regions, it is found more frequently than the ‘Oberösterreichischer Brünnerling’ [17]. Literature reference on this cultivar are very scarce and reflect general characteristics of the ‘Brünnerling’-cultivars. The fruit look similar to ‘Oberösterreichischer Brünnerling’ with oblate fruit [4,9]. ‘The ‘Welschbrunner’ is only found in a single accession in the GFG. The cultivar is triploid and groups with MUNQ 8105 [11].

## References

[1] H.-J. Bannier, Auf der Spur der Brünnerlinge. Beitrag zur pomologischen Klärung einer der wichtigsten Sortengruppen des Alpenraums., Pomologen-Verein e.V. Jahresheft 2024 (2025).

[2] W.H. von Hohberg, D. von Neuberg, M. Küsel, Georgica Curiosa Aucta, Umständlicher Bericht und klarer Unterricht Von dem vermehrten und verbesserten Adelichen Land- und Feldleben auf alle in Teutschland übliche Land- und Hauswirthschafften gerichtet... Nürnberg, 1701.

[3] R. Stoll, Oesterreichisch-Ungarische Pomologie, Zweite vermehrte Ausgabe. ed., Stoll, Rudolf, Klosterneuburg bei Wien., 1888.

[4] J. Löschnig, H.M. Müller, H. Pfeiffer, Empfehlenswerte Obstsorten (Normalsortiment für Niederösterreich), Komissionsverlag von Wilhelm Frick, Wien, 1912.

[5] G. Pfau-Schellenberg, Schweizerische Obstsorten, 2. Aufl. 1896 Aarau ed., Schweizerischer Landwirtschaftlicher Verein, St. Gallen / Zürich, 1863.

[6] T. Zschokke, Schweizerisches Obstbilderwerk., Wädenswil / Zug, Schweiz. Obst- und Weinbauverein, Verband Schweiz. Obsthandels- und Obstverwertungsfirmen, Verband Schweiz, Bern, 1925.

[7] E. Lucas, J.G.C. Oberdieck, Illustrirtes Handbuch der Obstkunde, Eugen Ulmer, Stuttgart, 1875.

[8] B. Kajtna, M. Schmidthaler, M. Suanjak, Kleiner Brünnerling. <https://www.arche-noah.at/media/obstsortenblatt_2025_kleinerbruennerling_web.pdf>, 2025 (accessed 14.05.2025.

[9] UPOV - INTERNATIONAL UNION FOR THE PROTECTION OF NEW VARIETIES OF PLANTS, APPLE: Guidelines for the conduct of tests for distinctness, uniformity and stability, UPOV Code(s): MALUS_DOM, Geneva TG/14/10 (2023). <https://doi.org/https://www.upov.int/edocs/tgdocs/en/tg014.pdf>.

[10] H.-J. Bannier, W. Schuricht, Pomologischer Abschlussbericht: Zweite pomologische Bestimmung der Apfelsorten der Deutschen Genbank Obst, in: O.b.o.F.O.f.A.a. Food (Ed.) Funding code: 2816BE007, <https://www.deutsche-genbank-obst.de/files/index>, 2021.

[11] L. Broschewitz, H.-J. Bannier, S. Reim, H. Flachowsky, M. Höfer, Microsatellite/SSR dataset: pomological and molecular characterization of apple cultivars (Malus × domestica Borkh.) of the German Fruit Genebank, Version 1, (2025). <https://doi.org/10.5073/20250813-152720-0>.

[12] J. Löschnig, Österreichische Gebietsapfelsorten, Obst und Garten (Heft 10) (1948) 218 ff.

[13] J. Löschnig, Österreichische Gebietsapfelsorten, Obst und Garten (Heft 9) (1948) 194 ff.

[14] J. Müller, O. Bißmann, e. al., Deutschlands Obstsorten, Eckstein & Stähle, Stuttgart, 1905-1933.

[15] B. Kajtna, M. Schmidthaler, M. Suanjak, Böhmischer Brünnerling. <https://www.arche-noah.at/media/obstsortenblatt_2025_boehmischerbruennerling_web.pdf>, 2025 (accessed 14.05.2025.

[16] E. Arming, B. Kajtna, M. Friedler, E. Silfverberg-Dilworth, H. Hueber, Obst-Inventur Österreich. Genetische Charakterisierung unserer Obstsammlungen, ARCHE NOAH unpubl. final report (2024).

[17] B. Kajtna, C. Wegenschimmel, M. Schmidthaler, Oberösterreichischer Brünnerling. <https://www.arche-noah.at/media/oberoesterreichischer_bruennerling.pdf>, 2025 (accessed 14.05.2025.
